# The Promoting Role of Different Carbon Allotropes Cocatalysts for Semiconductors in Photocatalytic Energy Generation and Pollutants Degradation

**DOI:** 10.3389/fchem.2017.00084

**Published:** 2017-10-31

**Authors:** Weiwei Han, Zhen Li, Yang Li, Xiaobin Fan, Fengbao Zhang, Guoliang Zhang, Wenchao Peng

**Affiliations:** School of Chemical Engineering and Technology, Tianjin University, Tianjin, China

**Keywords:** carbon allotropes, semiconductor, photocatalysis, cocatalysts, energy generation, pollutants degradation

## Abstract

Semiconductor based photocatalytic process is of great potential for solving the fossil fuels depletion and environmental pollution. Loading cocatalysts for the modification of semiconductors could increase the separation efficiency of the photogenerated hole-electron pairs, enhance the light absorption ability of semiconductors, and thus obtain new composite photocatalysts with high activities. Kinds of carbon allotropes, such as activated carbon, carbon nanotubes, graphene, and carbon quantum dots have been used as effective cocatalysts to enhance the photocatalytic activities of semiconductors, making them widely used for photocatalytic energy generation, and pollutants degradation. This review focuses on the loading of different carbon allotropes as cocatalysts in photocatalysis, and summarizes the recent progress of carbon materials based photocatalysts, including their synthesis methods, the typical applications, and the activity enhancement mechanism. Moreover, the cocatalytic effect among these carbon cocatalysts is also compared for different applications. We believe that our work can provide enriched information to harvest the excellent special properties of carbon materials as a platform to develop more efficient photocatalysts for solar energy utilization.

## Introduction

Environmental pollution and fossil fuels depletion are the most serious social problems nowadays. Since the discovery of the photocatalytic splitting of water on TiO_2_ electrodes by Fujishima and Honda in 1972, photocatalysis technology has become one of the most promising technologies for energy generation and environment remediation (Fujishima and Honda, 1972). Moreover, solar energy is clean, sustainable, and inexhaustible, which is therefore the most hopeful resource to solve the energy and environment problems (Chen et al., 2010a). Mostly, photocatalysis is a semiconductor-mediated process (Chen et al., 2010b; Wang et al., 2014; Zhang et al., 2016b). So far, kinds of semiconductor materials, including metal oxides, metal sulfides and metal containing salts have been used as photocatalysts. Some metal free materials, such as silicon, sulfur, graphic carbon nitride (g–C_3_N_4_), have also been developed as photocatalysts for the utilization of sunlight (Peng et al., 2013; Cao and Yu, 2014; Devi and ArunaKumari, 2014; He et al., 2015). However, some fundamental problems must be resolved before their real application, which are (1) low utilization efficiency of solar energy; (2) poor quantum efficiency; (3) severe photo corrosion (Zhang and Guo, 2013; Chowdhury and Balasubramanian, 2014; Han et al., 2015; Xie et al., 2015; Liu Y. et al., 2017b). To address these obstacles, modification of semiconductors with suitable cocatalysts is a frequent and effective solution (Yang J. H. et al., 2013). Metal nanoparticles and their compounds, especially noble metal based materials, are always used as cocatalysts (Bai et al., 2014; Zhang et al., 2015a; Zhong et al., 2016). Although they are effective to enhance the photocatalytic activity, the high cost and rare storage on earth limit their practical application (Ran et al., 2014). To develop cheap, highly efficient alternatives to replace noble metal based cocatalysts is still a great challenge in the photocatalysis filed.

**Figure 1:**
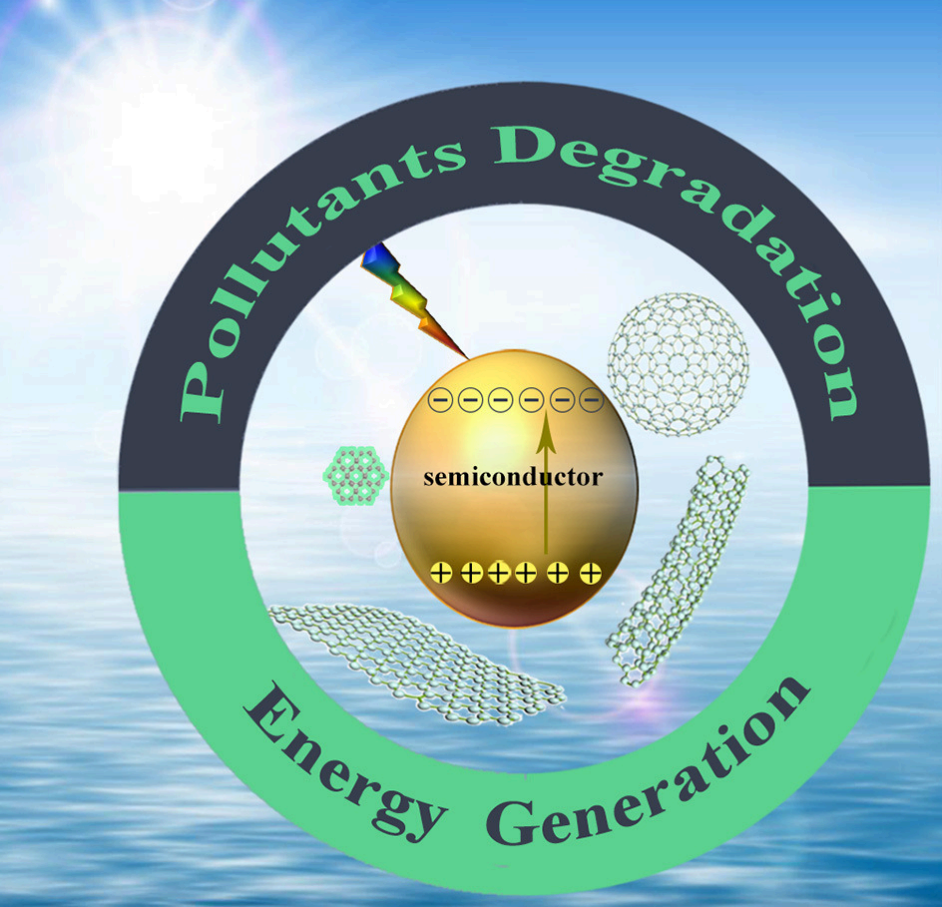
Schematic illustration of the photocatalytic applications of carbon materials based semiconductor composites.

Recently, carbon materials, including activated carbon (AC), fullerenes (C_60_), carbon nanotubes (CNTs), graphene (GR), and other carbon allotropes, have been widely investigated as cocatalysts for semiconductors in photocatalysis (Xiang et al., 2012; Ouzzine et al., 2014; Cao and Yu, 2016; Paulo et al., 2016; Yu et al., 2016). Specially, CNTs and GR have large specific surface areas (SSAs), excellent electric conductivity, high mechanical strength, and good thermal, and chemical stability, making them ideal substitute for noble metal cocatalysts (Zhang et al., 2012; Wang et al., 2013, 2017; Di et al., 2015; Han et al., 2016). Figure 1 shows the structure models of the carbon materials and their photocatalytic applications as cocatalysts simply. Many kinds of carbon cocatalysts based composites have been reported for photocatalytic reactions, and the cocatalytic mechanisms have also been discussed (Woan et al., 2009; Chen et al., 2011; Lee W. J. et al., 2012; Xie et al., 2013; Shearer et al., 2014; Li et al., 2015). For example, a graphene–TiO_2_ NPs hybrid was successfully synthesized by wrapping amorphous TiO_2_ NPs with GO using a one-step hydrothermal method by Lee and coworkers (Lee J. S. et al., 2012). The hybrid exhibited superior photocatalytic activity for the photodegradation of MB under the visible light irradiation. Fan et al. prepared a novel 3D AgX/graphene aerogels (X = Br, Cl) structured composite, which exhibited excellent photocatalytic and cycling performance for the degradation of MO and reduction of Cr^VI^ (Fan Y. et al., 2015). They also investigated photocatalytic enhancement mechanism of the graphene aerogels in the composite, which could effectively suppress the recombination of photogenerated holes, and electrons as a capable substrate for the photocatalyst. Tian et al. reported a new CQDs/hydrogenated TiO_2_ (H-TiO_2_) photocatalyst by assembling CQDs on the surface of H-TiO_2_ (Tian et al., 2015). The photocatalytic activity of CQDs/H-TiO_2_ was superior to P25, TiO_2_ nanobelts, and H-TiO_2_ nanobelts for the degradation of MO under UV-visible-NIR irradiation. The CQDs have excellent photo-induced electron transfer and reservoir properties, which could convert NIR light to visible light to be in full used by H-TiO_2_ and effectively suppress the recombination of electron-hole pairs. Generally, loading carbon materials as cocatalysts for semiconductors, the synergistic effect between them can increase the active sites, widen the absorption range of the solar light, facilitate the separation of the electron-hole pairs, and thus enhancing the photocatalytic activity.

To develop carbon materials based composite has attracted great attention for low cost and highly active photocatalysts. Lots of researches have been done on this subject, but a systematic summary about the key roles of different carbon allotropes as cocatalysts is still lacking. Herein, we aim to provide an overview on recent advances in the synthesis, multiple applications and mechanism of different carbon allotropes based composite photocatalysts. On behalf of this review, we wish more carbon based photocatalysts could be synthesized for environment remediation and energy generation.

## Photocatalysts synthesis

The synthesis process will affect the morphologies, properties and activities of the composite photocatalyts greatly. As shown in Table 1, we summarized the typical synthesis methods of the recently reported carbon based photocatalysts. The semiconductors could be loaded on carbon materials by one-step grinding, stirring, ultrasonic assisted dispersing or by some complicated multi-step synthesis methods. It can be concluded that mechanical mixing, hydrothermal/solvothemal, and sol-gel process are more frequently used. In addition, photocatalytic reduction and microwave-assisted method are also reported, and they may have a great potential due to the green and sustainable synthetic processes.

**Table 1:** Preparation methods and applications of carbon materials based semiconductor composites.

**Photocatalyst**	**Synthetic method**	**Photocatalytic applications**	**Light source**	**Reaction system (catalyst amount/solution)**	**Photocatalytic activity**	**References**
CdS−1D ZnO−2D GR	Two-step refluxing	Anaerobic reduction of 4-nitroaniline	300 W Xe lamp (λ ≥ 420 nm)	10 mg/40 ml (10 mg·L^−1^) with 40 mg HCOONH_4_	Conversion of 95% with high selectivity for PPD (> 98%) in 16 min	Han et al., 2015
TiO_2_/AC	Sol-gel	Oxidation of propene	UV lamp (radiation peaks at 257.7 nm or 365 nm)	–/100 ppmv [flow rates of 30 and 60 ml min^−1^ (STP)]	Conversion of nearly 60% for flow rate of 30 ml min^−1^	Ouzzine et al., 2014
Graphene–CNTs–CdS	Hydrothermal	Degradation of MB	Visible light irradiation	20 mg/50 ml (10 mg·L^−1^) MB solution	DP of ca. 40% in 30 min	Wang et al., 2013
CNT@TiO_2_	Solvothermal	Degradation of MO	300 W Xe lamp	50 mg/100 ml (15 mg·L^−1^) MO solution	8 times increment of the reaction rate compared to bare TiO_2_	Di et al., 2015
CNT–confined TiO_2_	Restrained hydrolysis	Degradation of MB	Xe lamp (λ ≥ 420 nm)	20 mg/50 ml (20 mg·L^−1^) organic pollutant solution	DP of 97.8% in 90 min	Chen et al., 2011
NCNT/TiO_2_ core/shell nanowires	Biomineralization followed by calcination	Degradation of MB or p-nitrophenol (PNP)	450 W Xe lamp (λ ≥ 420 nm)	Volume of 0.64 cm^2^/3.5 ml (10 ppm) MB or PNP solution	DP of ca. 100% in 4 h for MB	Lee W. J. et al., 2012
Graphene–wrapped TiO_2_ NPs	One-step hydrothermal treatment followed by calcination	Degradation of MB	450 W Xe lamp (λ ≥ 420 nm)	8 mg/8 ml (2.7 × 10^−2^ mM) MB solution	DP of ca. 90% in 1 h; rate constant k = 3.41 × 10^−2^ min^−1^	Lee J. S. et al., 2012
CQDs/hydrogenated TiO_2_ nanobelts	Oil bath reflux	Degradation of MO; hydrogen evolution	UV source: 350 W mercury lamp (254 nm); visible light source: 300 W Xe lamp; NIR light source: 250 W infrared lamp (λ < 760 nm)	20 mg/20 ml (20 mg·L^−1^) MO solution; 50 mg (with 1wt% Pt)/100 ml aqueous solution containing methanol (20% v/v)	DP of > 86%, 50% in 25 min under UV light, visible light irradiation, respectively; DP of 32% in 120 min under NIR light irradiation; 7.42 mmol h^−1^g^−1^	Tian et al., 2015
Graphene/ZnO	Hydrothermal	Degradation of deoxynivalenol	UV light (254 nm, 365 nm)	25 mg/50 ml (15 ppm) DON	DP of 99% in 30 min	Bai et al., 2017
Carbon nanotube–SiC	*In situ* growth	H_2_ evolution	300 W Xe lamp (λ ≥ 420 nm)	50 mg/100 ml of 0.1 M Na_2_S solution	R_H2_: 108 μmol h^−1^ g^−1^; 3.1 times higher than SiC	Zhou et al., 2015
BiVO_4_/CDs/CdS	Precipitation	Water splitting into H_2_ and O_2_	300 W Xe lamp (λ > 420 nm)	80 mg/100 ml ultrapure water	1.24 mol h^−1^	Wu et al., 2017
Graphite-like carbon spheres@TiO_2−x_	Two-step hydrothermal	H_2_ evolution; degradation of RhB, MB, CIP and 4-CP	UV-LEDs; 350 W Xe lamp (λ > 420 nm)	50 mg/80 ml (0.5 M) Na_2_S/Na_2_SO_3_ solution; 80 mg/80 ml (10 mg·L^−1^) pollutants solution	255.2 μmol h^−1^ g^−1^, 5.4 times higher than TiO_2−x_; 3.6/6.3 (RhB/MB) times higher than TiO_2_	Jiang et al., 2017
CdS NWs–CNT	Electrostatic self-assembly	Reduction of aromatic nitro organics	300 W Xe lamp (λ > 420 nm)	10 mg/40 ml (20 mg·L^−1^)	Nearly complete reduction of 4-NA in 5min	Weng et al., 2014
RGO–CdS	Microwave-assisted hydrothermal	Reduction of CO_2_	300 W Xe lamp (λ ≥ 420 nm)	100 mg/0.25 ml (4 M HCl And 0.12 g NaHCO_3_)	2.51 μmol h^−1^ g^−1^ QE: 0.8% at 420 nm	Yu J. et al., 2014
GR–CdS	Solvothermal	Selective reduction of aromatic nitro compounds	300 W Xe lamp (λ ≥ 420 nm)	10 mg/30 ml (20 mg·L^−1^) with 20mg ammonium oxalate	Conversion of almost 80% for 4-NA	Liu et al., 2014
A-Fe_2_O_3_/graphene	Hydrothermal	Degradation of RhB	350 W Xe lamp	30 mg/30 ml (10 mg·L^−1^) RhB solution with 0.7 ml H_2_O_2_ (≥ 30 wt%)	DP of 98% in 20 min	Han et al., 2014
MWCNT–TiO_2_ sphere	Hydrothermal	Degradation of gaseous styrene	365 nm UV-LED spot lamp	100 mg/25 ± 1.5 ppmv gaseous styrene	DP of 55.4% in 180 min	An et al., 2012
AC/Bi_2_WO_6_	Hydrothermal	Degradation of RhB	300 W Ultra-Vitalux lamp	250 mg/250 ml (10ppm) RhB	Totally degraded in 30 min	Murcia-Lopez et al., 2013
Carbon dots/g-C_3_N_4_/ZnO	Impregnation-thermal	Degradation of tetracycline (TC)	Xe lamp (λ ≥ 420 nm)	50 mg/100 ml (10 mg·L^−1^) RhB solution	DP of almost 100% in 30 min	Guo et al., 2017
CNT/Ag_3_PO_4_	Ultrasound followed by stir	Degradation of RhB	300 W Xe lamp (λ > 400 nm)	75 mg/75 ml (10 mg·L^−1^) TC solution	DP of ca. 10% in 12 min	Xu et al., 2014
TiO_2_/C_60_	Sonication followed by light irradiation	Degradation of MB and 4-CP	84W light sources (λ > 420 nm)	17 mg/25 ml (144 μM) MB; 15 mg/15 ml (10 mg·L^−1^) 4-CP	DP of 47% for MB and 82% for 4-CP in 40 min; 2 and 5 times of rate constant values of the bare TiO_2_	Mukthar Ali and Sandhya, 2014
GO–CdS	Two-phase mixing	degradation of various water pollutants and disinfection	Solar light simulator (λ ≥ 420 nm)	20 mg/50 ml (20 mg·L^−1^) water pollutants solution	DP of over 80% for AO7; nearly 100% of both *E. coli* and *B. subtilis* were killed in 25 min	Gao et al., 2013
CdS/GO	Solvothermal	H_2_ evolution	300 W Xe lamp (λ > 420 nm)	50 mg/100 ml of 1.25 M (NH4)_2_SO_3_ solution	1470 μmol h^−1^	Hong et al., 2015
TiO_2_/MWCNTs and TiO_2_/AC	Sol-gel	Degradation of Acid Blue 92	125 W high-pressure mercury lamp	60 ppm/20 ppm AB92	2 times of TiO_2_/MWCNTs faster than TiO_2_/AC in 120 min	Zarezade et al., 2011
CNTs/TiO_2_	Sol-gel	Degradation of MB	three UV-A lamps	20 mg/200 ml (10 mg·L^−1^)	DP of ca. 45% in 180 min	Li Z. et al., 2011
GO–TiO_2_ NFs	Sol-gel	Photocatalytic H_2_ evolution; dye-sensitized H_2_ evolution	300 W Xe lamp (λ > 320 nm); (420 nm)	0.5 g·L^−1^/ 10 vol% methanol aqueous solution; [RuL_3_] = 10μM, [EDTA]_0_ = 10 mM	The photocatalytic hydrogen production and photocurrent generation increased by 1.7 and 8.5 times	Kim et al., 2014
LaFeO_3_-rGO	High temperature sol-gel	Oxidation of MB or RhB	300 W Xe lamp (λ > 400 nm)	10 mg/100 ml (0.5 mg·L^−1^) MB solution or (1.25 mg·L^−1^) RhB solution	DP of ca. 98% in 70 min for MB	Ren et al., 2016
ZnS–rGO	Microwave irradiation	Degradation of MB and RhB	250 W tungsten halogen lamp	50 mg·L^−1^/ 0.1 mM dye solution	DP of 55.23% for MB and 90.37% for RhB in 120 min	Thangavel et al., 2016
Graphene/Cu_2_O	CVD method	Degradation of MO	300 W Xe lamp	20 mg/80 ml (30 mg·L^−1^) MO solution	DP of ca. 80% in 30 min	Zhang et al., 2016a
CdS–GR (RGO, SEG)	Solvothermal	Selective oxidation of benzyl alcohol in water	300 W Xe lamp (760 > λ > 420 nm)	8 mg/1.5 ml alcohol oxygen-saturated ultrapure water with 0.1 mmol alcohol	Conversion of ca. 35% for benzyl alcohol; the selectivity of ca. 72% for benzaldehyde	Zhang et al., 2013a
Ag@AgBr/CNT	Deposition-precipitation	CO_2_ reduction	150 W Xe lamp (λ > 420 nm)	500 mg/100 ml (0.2 M) KHCO_3_ solution	30 μmol h^−1^ g^−1^ for methane	Abou Asi et al., 2013
PSGM/rGO/CdS	Hydrothermal	H_2_ evolution	300 W Xe lamp (λ > 400 nm)	100 mg/100 ml (0.5 M) Na_2_S/Na_2_SO_3_ solution	175 μmol h^−1^; QE: 3.99% at 420 nm	Xu et al., 2016
RGO/InGaZn	Hydrothermal	H_2_ evolution	125 W Hg visible lamp (λ > 400 nm)	50 mg/50 ml (10 vol% CH_3_OH)	435.4 μmol h^−1^	Martha et al., 2014
(CNT–TiO_2_) _ox_	One-pot oxidation	H_2_ evolution	150 W mercury vapor lamp	170 mg/170 ml (10 vol% methanol or 0.02 M saccharide)	292.5 μmol h^−1^	Silva et al., 2015
CQDs/P25	Hydrothermal	H_2_ evolution	500 W halogen lamp (λ > 450 nm)	50 mg/25 ml (6.25 ml methanol)	9.1 μmol h^−1^ under UV-Vis light irradiation; 0.5 μmol h^−1^ under visible light irradiation	Yu H. et al., 2014
SWCNTs/TiO_2_	Hydrolysis	Degradation of organic pollutants	17 W mercury arc lamp (λ = 254 nm); 1500 W Xe lamp (700 > λ > 320 nm)	50 mg/500 ml of organic pollutants solution	Comparable degradation rates regarding Degussa P25 under UV irradiation	Murgolo et al., 2015
Ag_3_PO_4_-MoS_2_/graphene	Two-step hydrothermal	Degradation of phenols	500 W Xe lamp (λ > 420 nm)	20 mg/50 ml (20 mg·L^−1^) DCP solution	Nearly completed in 20 min, 60 min under simulated solar light, visible light irradiation	Peng et al., 2014
CQDs/ZnS	Hydrothermal and bath reflux	Degradation of MB, RhB, CIP	300 W Xe lamp (λ > 380 nm)	30 mg/50 ml (20 mg·L^−1^) for MB, RhB; 50 ml (10 mg·L^−1^) for CIP	Degradation rate is 1.67 and 2.11 times higher than ZnS for MB and RhB; DP is more than ZnS for CIP	Ming et al., 2016
C_60_@a–TiO_2_	Solution phase method	degradation of MB	8 W medium-pressure mercury lamp	100 mg/250 ml (5 mg·L^−1^) MB solution	Nearly completed in 60 min	Qi et al., 2016
GO–TiO_2_ CNT–TiO_2_	Liquid phase deposition	Degradation of Microcystin-LA	300 W Xe lamp; two 15 W fluorescent lamps (λ > 420 nm)	5 mg/10 ml (0.2 μM) MC-LA solution	DP of 100% in 5 min under solar light irradiation; DP of 88% in 2 h under visible light irradiation	Sampaio et al., 2015
CdS–cluster-decorated graphene	Solvothermal	H_2_ evolution	350 W Xe lamp (λ ≥ 420 nm)	20 mg/80 ml (8 ml lactic acid) mixed solution	1.12 mmol h^−1^ QE: 22.5% at 420 nm	Ye et al., 2012
GO–Ta_2_O_5_ CNT–Ta_2_O_5_	Hydrothermally assisted sol-gel	H_2_ evolution	High pressure Hg lamp	50 mg/no mentioned	1,600 μmol h^−1^ for CNT–Ta_2_O_5_; 140 μmol h^−1^ for GO–Ta_2_O_5_	Cherevan et al., 2014
TiO_2_-GR	Hydrothermal	Gas-phase degradation of benzene	Four 4W UV Lamps (254 nm)	300 mg/20 ml min^−1^ (250 ppm) benzene	Conversion of 6.4%; average mineralization ratio of 76.2%	Zhang et al., 2010
AgSiOx@CNT AgSiOx@RGO	In suit one-step	Degradation of MB	300 W Xe lamp (780 > λ > 400 nm)	50 mg/50 ml (50 ppm) of MB solution	Completed in 10 min by AgSiOx@CNT; completed in 7 min by AgSiOx@RGO	Jing et al., 2017
CDs/ZnIn_2_S_4_	Hydrothermal	Degradation of MO	300 W Xe lamp (λ ≥ 420 nm)	50 mg/100 ml (10 mg·L^−1^) dye solutions	DP of 100% in 40 min, 2.34 times higher than ZnIn_2_S_4_	Shi et al., 2017
CdS–carbon (C_60_, CNT, and GR)	Solvothermal	Selective oxidation of alcohols	300 W Xe lamp (λ ≥ 420 nm)	8 mg/1.5 ml oxygen-saturated BTF (0.1 mmol alcohol)	Conversion of 40%, 61% and 42% along with 100% selectivity over CdS–RGO, CdS–C_60_ and CdS–CNT in 3 h	Zhang et al., 2013b
CNT/Cd0.1Zn0.9S	Hydrothermal	H_2_ evolution	300 W Xe lamp (λ ≥ 420 nm)	50 mg/80 ml (0.35 M Na_2_S and 0.25 M Na_2_SO_3_) aqueous solution	1,563.2 μmol h^−1^ g^−1^; QE: 7.9%	Yu et al., 2012
TiO_2_/graphene aerogels (GAs)	Hydrothermal	Degradation of MO	300 W Xe lamp	no mentioned/70 ml (10 mg·L^−1^) MO solutions	DP of 90% in 5 h	Qiu et al., 2014

### Hydrothermal/solvothemal methods

Hydrothermal or solvothermal methods are the most frequently used ways due to their mild reaction conditions, high product purity, controllable morphology, good crystallinity, and uniform distribution of obtained products (Li Q. et al., 2011). For example, Liu et al. synthesized GR–CdS nanocomposites by an one-step solvothermal method using DMSO as reductant and sulfure source (Liu et al., 2014). In the preparation procedure, GO was dispersed in DMSO to obtain the GO–DMSO dispersion, Cd(CH_3_COO)_2_·2H_2_O was then added. The mixture was then treated at 453 K for 12 h to obtain the final composites. The photocatalytic activity of GR–CdS nanocomposites for selective reduction of aromatic nitro compounds was dramatically enhanced compared to the pure CdS. This can be ascribed to the synergistic effect with graphene addition, the increased visible light absorption range and intensity, the improved lifetime and charge transfer ability, and the enhanced adsorption capacity of this nanocomposite toward the nitro compounds.

Han et al. synthesized 2D hexagonal α-Fe_2_O_3_/graphene nanoplate composites by a simple one-step hydrothermal method with no template (Han et al., 2014). Using hydrothermal method, not only the effective reduction of the GO to graphene was achieved, but intimate contact was also formed between the α-Fe_2_O_3_ nanoplates and graphene. A significant enhancement for photocatalytic degradation of RhB could be observed after the combination with graphene cocatalyst. An et al. fabricated MWCNT–TiO_2_ sphere composites by a facile one-step hydrothermal method using TiF_4_ as titanium source and CNTs as structure regulator (An et al., 2012). The effects of hydrothermal temperature and hydrothermal time on the structural characteristics of MWCNT–TiO_2_ photocatalysts were investigated. Decreasing hydrothermal temperature or prolonging the hydrothermal time could lead to the enhancement of the photocatalytic degradation efficiency of both gaseous (i.e., styrene) and aqueous (i.e., MO) phase. Decreasing the hydrothermal temperature could lead to the crystallite size decrease of TiO_2_ (Table 2), while prolonging the hydrothermal time will increase the synergistic effects between TiO_2_ and MWCNTs, thus promoting the photocatalytic performance.

**Table 2:** Crystallite size of TiO_2_ in Pure TiO_2_ and MWCNT–TiO_2_ photocatalysts.

**Samples prepared under different conditions**	**Crystallite size (nm)**
Pure TiO_2_	44.7
7.2 wt % MWCNTs	33.1
18.9 wt % MWCNTs	30.1
31.7 wt % MWCNTs	29.9
48.2wt % MWCNTs	30.6
51.6 wt % TiO_2_	23.2
68.4 wt % TiO_2_	26.7
81.1 wt % TiO_2_	30.1
89.6 wt % TiO_2_	35.2
120°C	24.2
150°C	26.7
180°C	27.3
210°C	28.4
24 h	25.5
48 h	26.5
72 h	26.7

*Reprinted from An et al. (2012), Copyright 2012, with permission from American Chemical Society*.

As reported by Murcia-López et al. the calcination could be applied after hydrothermal process to prepare the AC/Bi_2_WO_6_ and AC/TiO_2_/Bi_2_WO_6_ photocatalysts (Murcia-Lopez et al., 2013). The introduction of optimized percentage of AC (2 wt%) could form 3D-hierarchical structures of both AC/Bi_2_WO_6_ and AC/TiO_2_/Bi_2_WO_6_, which exhibited improved photocatalytic activities for the RhB degradation under both UV-vis and visible illumination compared to pure Bi_2_WO_6_. Here, the presence of AC could stimulate the 3D-hierarchical structure formation, and will increase the surface area and absorption ability of the catalyst at the same time.

### Mechanical mixing method

The loading of carbon cocatalysts can also be performed by simple mechanical mixing processes, such as magnetic stirring, ball milling, and ultrasonication (Xu et al., 2014; Guo et al., 2017). Ali et al. used C_60_ as cocatalysts for the modification of TiO_2_ using a simple sonication assisted mixing method (Mukthar Ali and Sandhya, 2014). The C_60_ molecules were first dispersed in β-cyclodextrin (CD), and then mixed with the suspension of TiO_2_ with the assistance of sonication under sunlight. According to the HRTEM images of the composites, C_60_ cocatalysts are dispersed in the composite without aggregation. They believed that the non-aggregated C_60_ cocatylysts played a key role in increasing the amount of reactive oxygen species (ROS) and suppressing photogenerated charge recombination, thus leading to the enhanced photocatalytic activity. The photocatalytic activity of the composite shows 2 and 5 times higher than the bare TiO_2_ for the degradation of MB and 4-CP, respectively. Gao and his coworkers successfully synthesized GO–CdS composites by a novel two-phase mixing method (Gao et al., 2013). By simply stirring for 24 h, the two phases are mixed into a homogeneous solution, and CdS nanoparticles are then uniformly deposited on GO sheets (see Scheme 1 in the original paper, Gao et al., 2013). The obtained composites show higher photocatalytic degradation and disinfection activities than CdS under visible light irradiation.

However, using the mechanical mixing method, the interaction force between semiconductors and carbon materials is a little weak without the formation of chemical bonds, resulting in a relatively lower activity enhancement compared to that from hydrothermal/solvothemal methods. For example, Hong and his coworkers reported that CdS/GO photocatalysts synthesized by *in situ* solvothermal method showed much higher H_2_ evolution activity than that synthesized by mechanical loading (Hong et al., 2015).

### Sol-gel method

The sol-gel method is another widely applied method to get a close chemical interaction between semiconductors and carbon cocatalysts (Zarezade et al., 2011; Morales-Torres et al., 2012; Ng et al., 2012). Generally, this method need to prepare the sol first, which is then mixed with the carbon materials uniformly. Subsequently, the gel is formed by aging followed with high temperature calcination to obtain the final composites. This method can control the crystal structure and uniformity of the supported nanoparticles, thus can fabricate photocatalysts with high activities. Li et al. used surfactant wrapping sol-gel method for the synthesis of CNT/TiO_2_ core-shell nanocomposites (Li Z. et al., 2011). Using this method, they prepared uniform and distinct nanoscale anatase TiO_2_ layer on the CNTs with tailored TiO_2_ layer thickness with different Ti precursors (TEOTi, TTIP, and TBT). The CNT/TiO_2_ composite prepared from TBT has thinner TiO_2_ layer that provides shorter traveling distance for electron transferring to the CNT core, the activity for the degradation of MB was therefore higher than those prepared from TEOTi and TTIP. Kim et al. prepared GO–TiO_2_ nanofibers (NFs) by using a sol-gel method and an electro-spinning technique (Kim et al., 2014). They also compared the activity of GO–TiO_2_ NF with GO(s)–TiO_2_ NF (prepared by covering GO sheets on external surface of TiO_2_ NF). Due to the stronger electronic coupling between GO and TiO_2_ matrix and the reduced light shielding effect by hiding GO inside of TiO_2_ NF, the photocatalytic H_2_ production of GO–TiO_2_ NF was higher than GO(s)–TiO_2_ NF.

Although the materials prepared by sol-gel method have high purity and uniform particle size, some problems still exist, such as relatively long reaction time, large shrinkage during drying, and easy sintering with high temperature calcination. For example, Ren et al. prepared nanostructured LaFeO_3_ nanoparticles (NPs) with rGO as a 2D template using a high temperature sol-gel method (Ren et al., 2016). Although the addition of C-support or rGO reduces the sintering degree of LaFeO_3_, it remains difficult to avoid sintering during the high temperature calcination for a long time.

### Other methods

Microwave-assisted method is a green synthesis method based on the characteristics of microwave heating with tremendous advantages (Tian et al., 2016). Preparation of catalysts with special structure and high yield would be finished in a very short time using microwave heating. Thangavel et al. prepared the ZnS–rGO nanohybrids via microwave irradiation for 20 s over two cycles (Thangavel et al., 2016). Interestingly, Raman spectrum of the hybrids indicates the complete reduction of GO into rGO via the microwave treatment. After 2 h of irradiation, the ZnS–rGO showed higher degradation efficiency for MB (about 55.23%) and RhB (about 90.37%) than that of bare ZnS (about 40.79% for MB and 56.56% for RhB), respectively. They attributed the high activity to tight intermolecular binding, good interfacial contact between ZnS and rGO in the hybrid, and enhanced charge-transfer properties of rGO in nanohybrid. Zhang et al. successfully synthesized the graphene/Cu_2_O composites by a CVD (chemical vapor deposition) method. They also investigated the effects of the CVD growth parameters on the graphene flakes. The obtained composites were effective for the photocatalytic methyl orange degradation (Zhang et al., 2016a).

## Photocatalytic applications

The photocatalytic activity of pure semiconductors can be enhanced by the addition of carbon materials as cocatalysts. The obtained composites are mainly used for the photocatalytic pollutants degradation, water splitting, CO_2_ reduction, organic synthesis and so on (Abou Asi et al., 2013; Zhang et al., 2013a; Colmenares et al., 2016; Li K. et al., 2016; Zeng et al., 2017). In the following sections, we will focus their applications for photocatalytic hydrogen evolution and pollutants degradation.

### Photocatalytic hydrogen evolution

**Figure 2:**
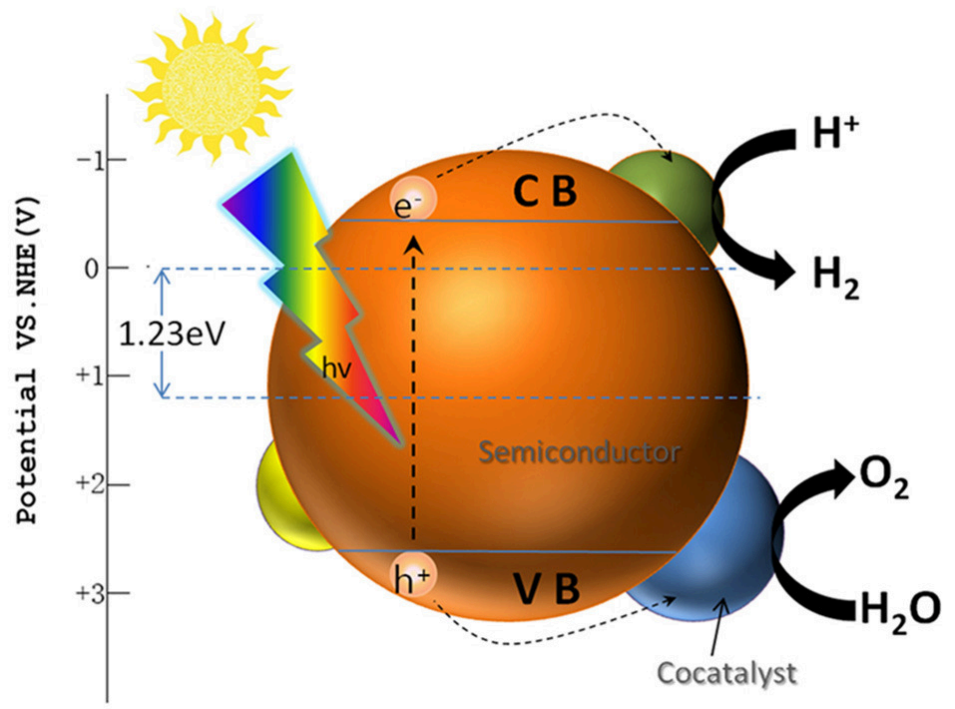
Fundamentals of semiconductor photocatalytic water splitting for hydrogen evolution.

Hydrogen is considered as one of the most potential alternative energy in the twenty-first century (Zhang et al., 2015b; Zou and Zhang, 2015). Among the present hydrogen production methods, photocatalytic water splitting driven by sustainable solar energy is an ideal way to achieve clean hydrogen production (Matsuoka et al., 2007; Wang et al., 2009; Hisatomi et al., 2014). Figure 2 describes the photocatalytic water splitting process with the presence of cocatalysts. Under the light irradiation, the electrons are photoexcited from the valence band (VB) to the conduction band (CB), while the holes are left in the VB, resulting in the separation of electrons and holes. Generally, for photocatalytic water splitting, the CB potential of semiconductor has to be more negative than hydrogen electrode potential EH^+^/H_2_, while the VB potential should be more positive than oxygen electrode potential EO_2_/H_2_O (Xu et al., 2016). Moreover, due to the impact of semiconductor band bending and presence of surface overpotential, the band gap of semiconductor should be larger than 1.23 eV to split water into H_2_ and O_2_ (Matsuoka et al., 2007; Moniz et al., 2015).

**Figure 3:**
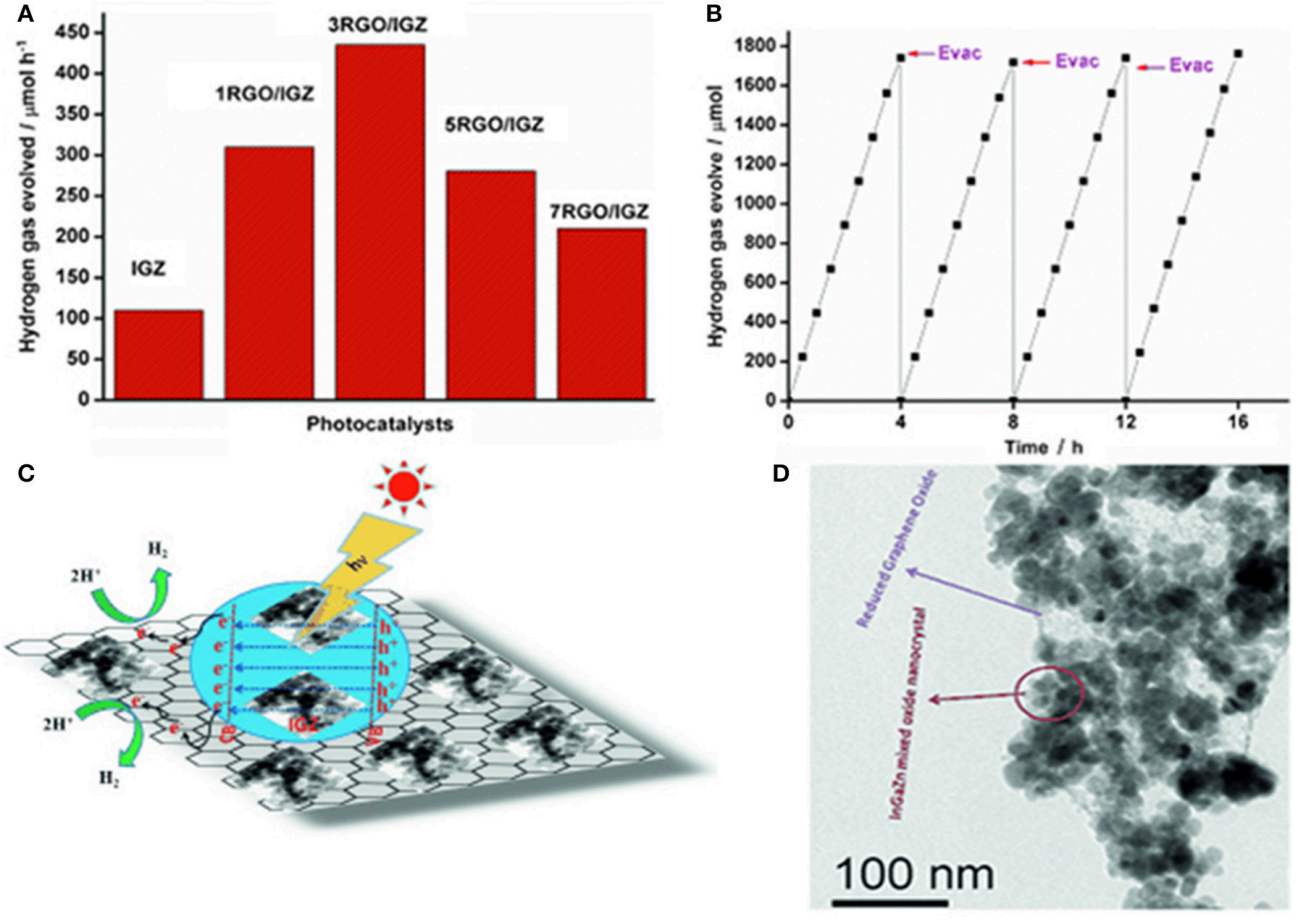
**(A)** Photocatalytic H_2_ evolution over IGZ, 1RGO/IGZ, 3RGO/IGZ, 5RGO/IGZ, and 7RGO/IGZ under visible-light irradiation; **(B)** Time course of H_2_ evolution over 3RGO/IGZ; **(C)** Mechanism of photocatalytic H_2_ composites; **(D)** TEM image of 3RGO/IGZ (Reprinted from Martha et al., 2014, Copyright 2014, with permission from Wiley-VCH).

Carbon materials are effective H_2_ evolution cocatalysts for the semiconductors mainly due to their large surface area and good charge mobility on their surface. Martha et al. synthesized RGO/InGaZn nanocomposites using a one-pot hydrothermal method (Martha et al., 2014). They also evaluated the effects of RGO percentage on the H_2_ evolution activity under visible-light irradiation (λ > 400 nm) (Figure 3). Three wt% rGO was proved to be the best loading percentage, and the H_2_ generation rate can be as high as 435 μmol/h (Figure 3A). As shown in Figure 3D, InGaZn was uniformly dispersed on the surface of RGO, which was beneficial for the electrons moving from InGaZn to RGO. Moreover, the RGO could also provide more active adsorption sites and photocatalytic reaction centers. The stability test of RGO/InGaZn composite was also tested, and no deactivation could be found after four recycles (Figure 3B).

Silva et al. combined TiO_2_ and CNTs using two different methods: hydration-dehydration labeled as (CNT_ox_-TiO_2_) and one-pot oxidation (labeled as (CNT–TiO_2_)_ox_) (Silva et al., 2015). One wt% Pt was then loaded followed by calcination at 473 K and 673 K, respectively. The optimized catalyst Pt/(CNT–TiO_2_)_ox_-473 could obtain a H_2_ evolution rate of 485 μmol/h, 2.4 times compared to the Pt/TiO_2_-473. According to the infrared attenuated total reflectance (ATR) spectra (see Figure 1 in the original paper, Silva et al., 2015), the bands from C = C and C–H are weaker in (CNT–TiO_2_)_ox_ than in CNT_ox_-TiO_2_, indicating a better dispersion of the TiO_2_ particles at the surface of CNT in (CNT–TiO_2_)_ox_. This conclusion can be further confirmed by SEM and TEM images in. The better photocatalysis performance of (CNT–TiO_2_)_ox_ might be related to the stronger interface interaction between TiO_2_ and CNT, which is promoted by the oxidative treatment according to the ATR analysis.

**Figure 4:**
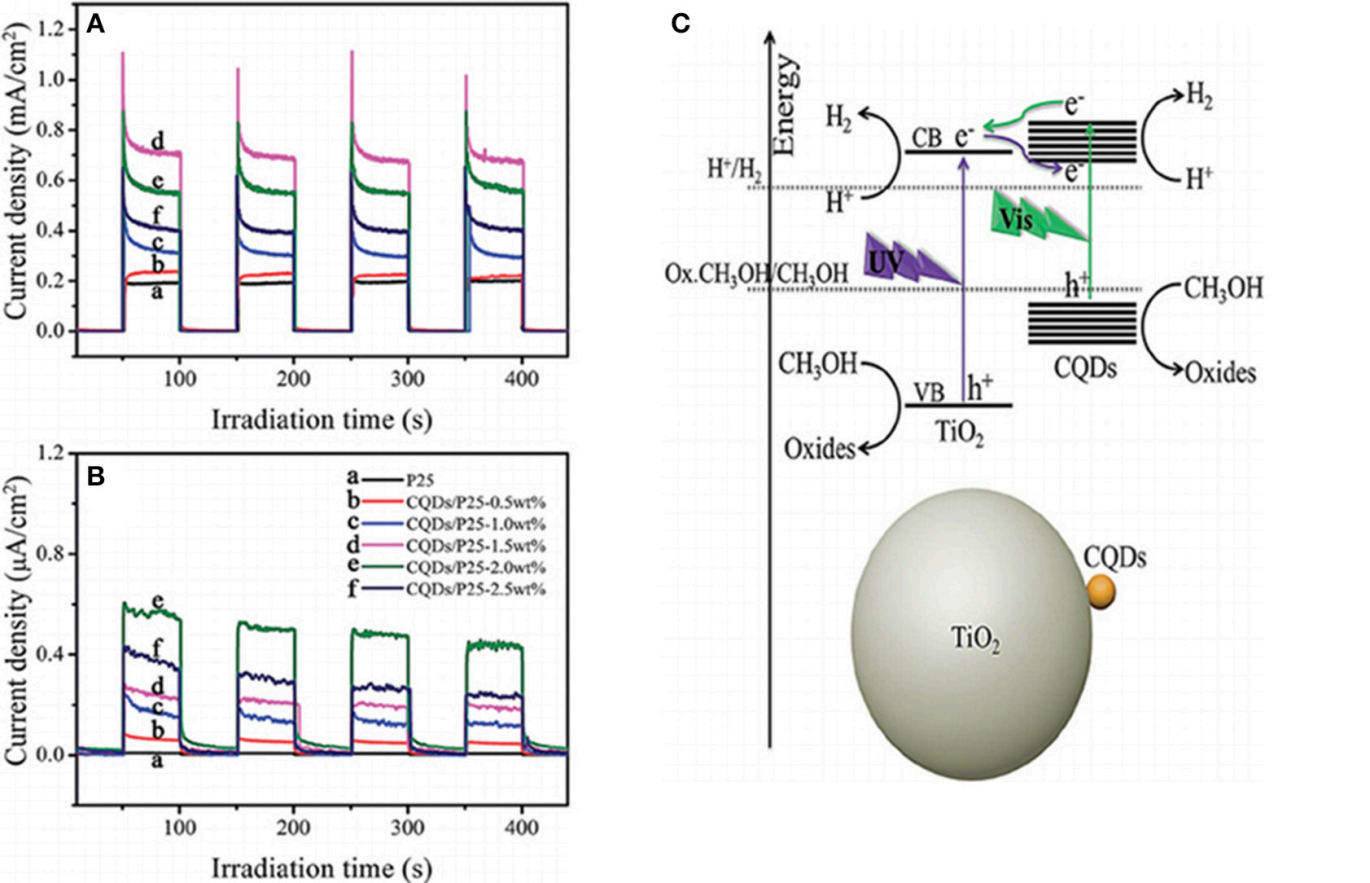
**(A)** Schematic illustration for the photocatalytic H_2_ production mechanism over the CQDs/P25 under UV-Vis and visible light (λ > 450 nm) irradiation; **(B,C)** The transient photocurrent response of P25 and the CQDs/P25 composites with different amount of CQDs in 1 M Na_2_SO_4_ aqueous solution under UV-Vis light and visible light (λ > 450 nm) irradiation (Reprinted from Yu H. et al., 2014, Copyright 2014, with permission from Royal Society of Chemistry).

Loading carbon materials as cocatayst, the bandgap of semiconductors could be narrowed to utilize the visible light with longer wavelength. Yu et al. prepared the CQDs/P25 composites with a “dyade”-like structure and applied them for photocatalytic hydrogen evolution under both UV-vis and visible light irradiation (Figure 4) (Yu H. et al., 2014). With methanol as the sacrificial agent, CQDs/P25–1.5 wt% showed the best photocatalytic performance under UV-vis light irradiation, and the evolution rate could reach 9.1 μmol/h, 4 times higher than that of pure P25 (2.3 μmol/h). While CQDs/P25–2.0 wt% was the optimized one under visible light with a H_2_ evolution rate of 0.5 μmol/h. The photocurrent response of these composites are shown in Figures 4A,B, which are consistent with the photocatalytic results. They believed that CQDs played dual roles to improve the photocatalytic activity of P25. CQDs could act as electron acceptors to improve the charge separation under UV-vis light irradiation. Meanwhile, they also served as a photosensitizer to sensitize P25 into a visible light response “dyade” structure for H_2_ evolution under visible light irradiation.

Heteroatom doped carbon materials, such as nitrogen doped graphene, are proved to be better cocatalysts for semiconductor photocatalysts in recent years (Putri et al., 2015). Yue et al. synthesized a ternary visible-light-driven photocatalyst for hydrogen evolution reaction. After decorating the CdS/Nb_2_O_5_ heterojunction structure with N-doped graphene (NGR) nanosheets (Yue et al., 2017), the hybrid photocatalyst (2 wt% NGR) exhibited a high H_2_ evolution rate of 100 μmol h^−1^ g^−1^, which was about 7.7 times than the pure CdS. Doping with nitrogen atom could change the electron density of the GR surface, thus can separation the photogenerated charges more efficiently. Jia et al. synthesized a series of nanocomposites by coupling CdS nanoparticles with NGR through calculation (Jia et al., 2011). The N-graphene/CdS was proved to be more efficient photocatalysts for hydrogen evolution compared to the CdS supported on undoped graphene. Significantly, the photocatalytic H_2_ evolution rate of the N-graphene (2 wt %)/CdS reached 210 μmol h^−1^ without the addition of metal cocatalyst, which was much higher than graphene/CdS (99 μmol h^−1^) and GO/CdS (95 μmol h^−1^) with the same percentage of cocatalysts.

### Photocatalytic degradation of pollutants

Photocatalytic degradation of pollutants is another important application of photocatalysts. Photocatalysts can adsorb and degrade pollutants in water and toxic gas in air under illustration, which thus has great potential for environmental remediation. Previous studies have shown that photocatalysis technology can not only degrade organic pollutants into CO_2_, H_2_O, and inorganic salt, but also eliminate the heavy metal ions (Akpan and Hameed, 2009; Peng et al., 2014; Murgolo et al., 2015; Jing et al., 2017).

**Figure 5:**
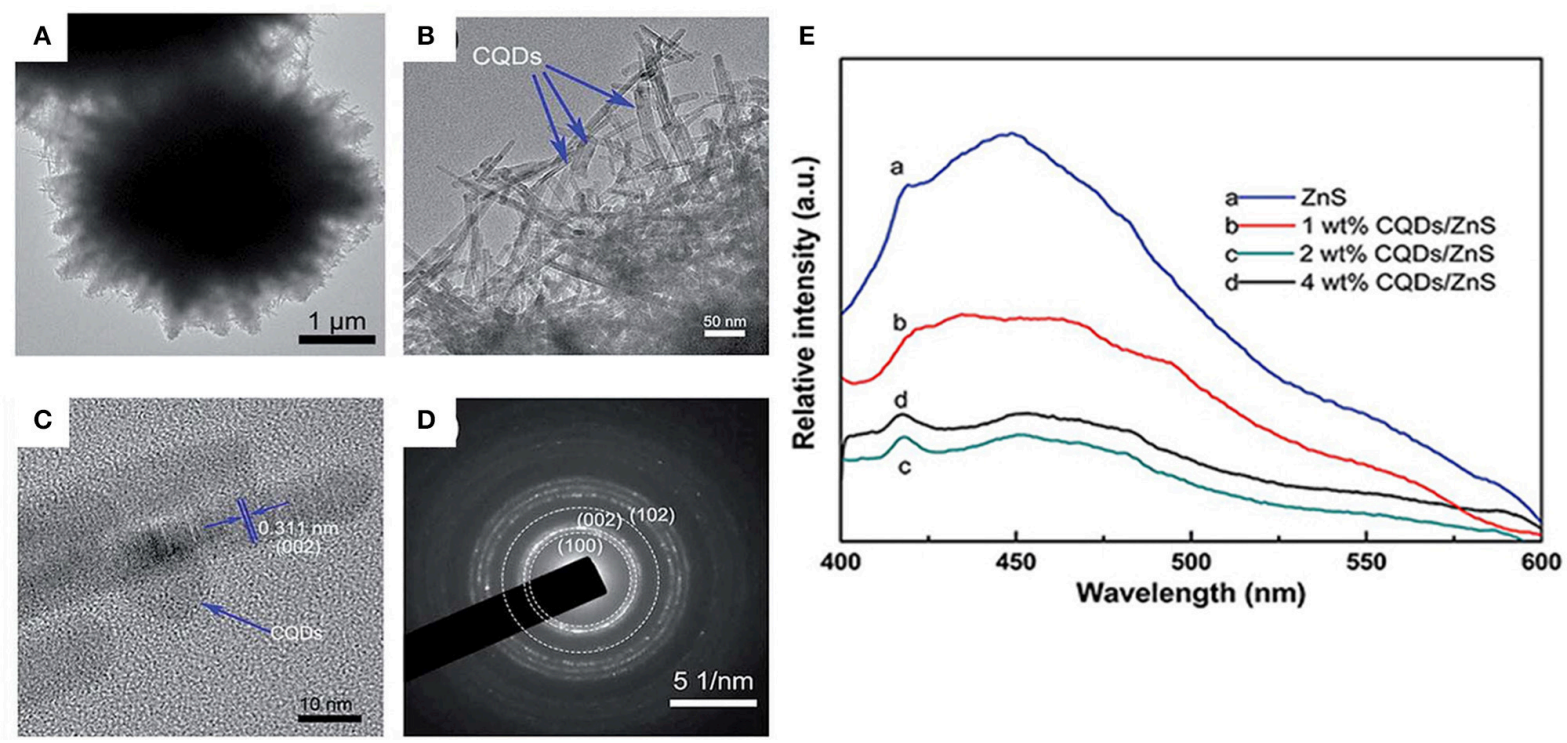
**(A,B)** TEM images of CQDs/ZnS hybrid materials; **(C)** HRTEM image of the CQDs/ZnS hybrid materials; **(D)** SAED of the dandelion-like ZnS; **(E)** PL spectra of pure ZnS and CQDs/ZnS hybrid materials (Reprinted from Ming et al., 2016, Copyright 2016, with permission from Royal Society of Chemistry).

Ming et al. synthesized dandelion-like ZnS/CQDs hybrid materials using hydrothermal method with CTAB as surfactant (Ming et al., 2016). As shown in Figures 5A–D, some dark dots are distributed on the ZnS nanowires uniformly. Coating the optimal content of 2 wt% CQDs, the photocatalyst showed the highest degradation rate, which was about 1.67 and 2.11 times higher than bare ZnS for MB and RhB, respectively. As illustrated in Figure 5E, the intensity of the PL emission band decreased obviously after the loading of CQDs on ZnS. The 2 wt% CQDs/ZnS possessed the lowest intensity, suggesting the lowest recombination possibility of photoexcited holes and electrons. They also proposed the photocatalytic mechanisms on the CQDs/ZnS hybrid:

(1) $$\begin{array}{l} \text{ZnS}+\text{hv}\to {{\text{e}}_{\text{ZnS}}}^{-}+{{\text{h}}_{\text{ZnS}}}^{+} \end{array}$$

(2) $$\begin{array}{l} {\text{e}}_{\text{ZnS}}^{-}\to {\text{e}}_{\text{CQDs}}^{-} \end{array}$$

(3) eCQDs−+O2→O*2−

(4) hZnS++H2O→O*H−

(5) $$\begin{array}{l} 2^{*}\text{OH}\to {\text{H}}_{2}{\text{O}}_{2} \end{array}$$

(6) $$\begin{array}{l} {\text{H}}_{2}{\text{O}}_{2}{+}^{*}{\text{O}}_{2}^{-}\to {\text{OH}}^{-}{+}^{*}\text{OH}+{\text{O}}_{2} \end{array}$$

(7) O*H+dye→H2O+CO2+intermediates

Qi and his co-workers prepared a series of fullerene-modified anatase TiO_2_ (C_60_@a-TiO_2_) nanocomposites by a simple solution phase method (Qi et al., 2016). By the introduction of C_60_, the activity of C_60_@a-TiO_2_ for photocatalytic degradation of MB could be enhanced greatly under UV-A light irradiation. In order to confirm the electronic structures of C_60_@a-TiO_2_, the density functional theory (DFT) was used for a theoretical calculation toward the C_60_-COOH@a-TiO_2_ (101) surface. The adsorption energy and the projected density of states (PDOS) for the C_60_-COOH@a-TiO_2_ (101) surface were calculated. Strong covalent interaction between C_60_ and the a-TiO_2_ (101) surface was present with the calculated adsorption energy of 3.61 eV. Moreover, the introduction of C_60_ narrows the band gap to 0.8 eV, resulting in the red shift of light absorption edge of the C_60_-COOH@a-TiO_2_ heterojunctions. According to the DFT results, there is an additional doping state present between the valance band and conduction band by the incorporation of C_60_ on the a-TiO_2_ (101) surface. The activity of C_60_@a-TiO_2_ is therefore enhanced with more efficient charge separation efficiency and increased light absorption range.

Sampaio et al. used both GO–TiO_2_ and CNT–TiO_2_ materials for the photocatalytic degradation of the cyanobacterial toxin, microcystin-LA (MC-LA) under simulated solar light and visible light irradiation (Sampaio et al., 2015). The GO–TiO_2_ composite containing 4 wt% of GO exhibited the highest photocatalytic activity under both simulated solar light and visible light irradiation. The enhanced activity of GO–TiO_2_ was attributed to the optimal assembly and interfacial coupling between TiO_2_ nanoparticles and GO sheets, which can effectively inhibit electron-hole recombination. While the activity of CNT–TiO_2_ for the MC-LA removal under visible light irradiation was mostly due to adsorption instead of photocatalytic degradation.

Murgolo et al. fabricated a composite photocatalyst by combining SWCNTs with nano-sized TiO_2_ NRs (Murgolo et al., 2015). The composite showed tailored photocatalytic properties for the photocatalytic degradation of a mixture of 22 organic pollutants under both UV and simulated solar light. The experiment results showed that this composite displayed comparable degradation rates over Degussa P25 under UV irradiation. While the SWCNTs/TiO_2_ showed slightly lower efficiency than Degussa P25 under simulated solar irradiation. The SWCNTs/TiO_2_ can be reused easily by a mild centrifugation or a filtration. This photocatalyst has proved to be a promising candidate in photocatalytic pollutants degradation, which can also be integrated with a biological step for the enhanced removal of emerging organic pollutants.

Heteroatoms doped carbon materials are also effective cocatalysts for photocatalytic degradation reaction. Liu et al. synthesized N-CNT/mpg-C_3_N_4_ composites via thermal polycondensation (Liu J. et al., 2017). N-CNT has better electronic conductivity and more defective structure than undoped CNT, which could therefore accept electrons more easily. Benefiting from the synergistic effect between N-CNT and mpg-C_3_N_4_, the composites show enhanced photo-degradation activity for rhodamine B, methyl orange and tetracycline hydrochloride under visible light irradiation. Due to the special 2D structure of graphene, which can also be combined with other layered materials to fabricate hybrid cocatalysts (Chen et al., 2017; Peng et al., 2017). Our group have used the MoS_2_/graphene hybrids for the modification of CdS and Ag_3_PO_4_, and the obtained composites showed improved photocatalytic activity for phenols degradation and nitroaromatic compounds detoxification (Peng et al., 2014, 2016). The photo-activity of the final composite could also be adjusted by changing the ratio of MoS_2_ and graphene.

## Comparison of carbon allotropes as cocatalysts

**Figure 6:**
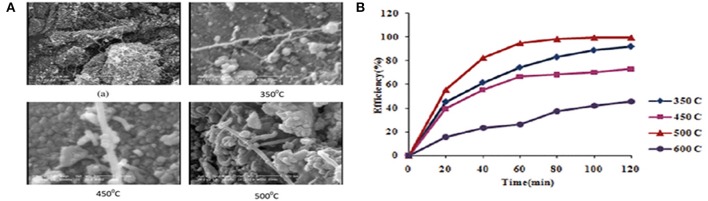
**(A)** SEM images of the **(A)** acid treated MWCNTs (1 mm), and TiO_2_/MWCNTs calcined at various temperatures; **(B)** Effect of calcination temperatures on photocatalytic activity of TiO_2_/MWCNTs (Reprinted from Zarezade et al., 2011, Copyright 2011, with permission from Royal Society of Chemistry).

There have been some other relevant reviews on this subject, but as far as we are concerned, a horizontal comparison of these carbon cocatalysts in photocatalysis field is still lack. In this section, we summarized some examples which compared different carbon cocatalysts for the modification of semiconductors. Zarezade et al. used sol-gel method to synthesize TiO_2_/AC and TiO_2_/MWCNT hybrid materials (Zarezade et al., 2011). Although the surface area of TiO_2_/MWCNTs was smaller than that of TiO_2_/ACs, the activity of TiO_2_/MWCNTs was even higher for photocatalytic degradation of AB92. The defects of MWCNTs could be used as anchor sites for the growth of TiO_2_ crystallites, which can lead to the uniform distribution of TiO_2_ on the MWCNT surface. After calcination of the composite at 500°C (Figure 6A), a remarkable photocatalytic performance could be achieved with a maximum degradation percentage of 86% in 2 h (Figure 6B).

**Figure 7:**
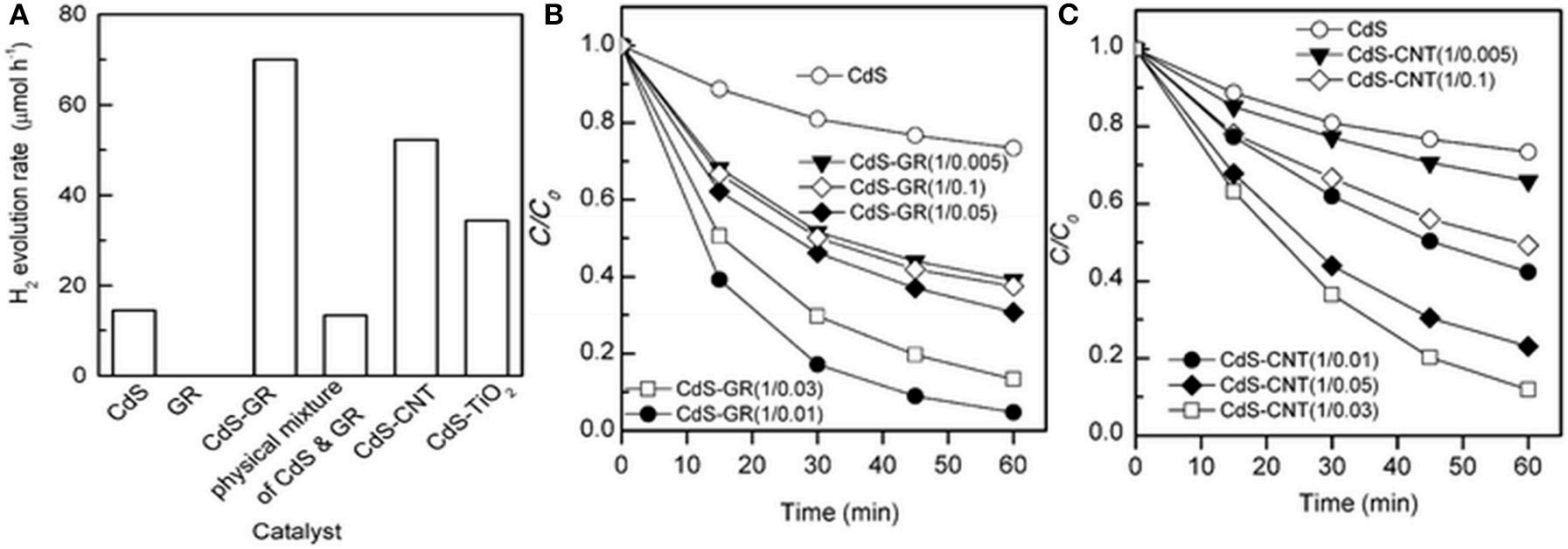
**(A)** Comparison of photocatalytic H_2_ evolution rate of different photocatalysts; **(B)** Photocatalytic degradation of MO over the CdS–GR and **(C)** CdS–CNT composites with different mass ratios of CdS: carbon material under visible-light irradiation (Reprinted from Ye et al., 2012, Copyright 2012, with permission from Royal Society of Chemistry).

Ye's group compared the photocatalytic behaviors of CdS–graphene (CdS–GR) and CdS–carbon nanotube (CdS–CNT) nanocomposites as photocatalysts for the hydrogen evolution and the degradation of methyl orange (MO) under visible-light irradiation (Ye et al., 2012). Figure 7A reveals that both the CdS–GR and the CdS–CNT composites display enhanced photocatalytic H_2_ evolution activities. Furthermore, the CdS–GR composite is more efficient than the CdS–CNT composite under their optimized mass ratios. The H_2_ evolution rate over the CdS–GR composite could reach 70 μmol h^−1^, which is 1.3 times higher than that of the CdS–CNT (52 μmol h^−1^). Similarly, Figure 7B shows that GR is more efficient to enhance the photocatalytic performance of CdS for the degradation of MO. The degradation percentage of MO over the optimized CdS–GR (1: 0.01) is as large as 95%, 1.8 times higher than that of the optimized CdS–CNT (1: 0.03) after 60 min irradiation (Figure 7C). The stronger interaction and larger contact interface between CdS and GR facilitate the transfer of photogenerated electrons from CdS to GR, leading to a higher efficiency in the separation of photogenerated electron-hole pairs and a higher photocatalytic performance of the CdS–GR composite than the CdS–CNT composite.

Cherevan and coworkers hybridized both multi-walled CNTs and graphene oxide (GO) with Ta_2_O_5_ semiconductor via a *in situ* hydrothermally assisted sol-gel method (Cherevan et al., 2014). Surprisingly, CNT–Ta_2_O_5_ hybrid exhibited superior performance over GO–Ta_2_O_5_ hybrid, and a maximum H_2_ evolution rate of 1,600 μmol h^−1^ could be obtained for CNT–Ta_2_O_5_. This result is opposite to many other studies, which could be attributed to two reasons: (1) the amount of Ta_2_O_5_ in the GO hybrid is much lower than in the CNT hybrid; (2) annealed CNTs are expected to possess better charge transfer properties than highly defective GO.

Jing et al. compared the degradation efficiencies of methylene blue (MB) over AgSiOx@CNT and AgSiOx@RGO nanocomposites under visible light (Jing et al., 2017). Interestingly, AgSiOx@CNT has a better photodegradation performance than AgSiOx@RGO at a small amount of CNTs, while the removal rate with AgSiOx@RGO is faster than AgSiOx@CNT at high carbon contents. This is probably because the different functional mechanism of these two carbon materials. The low content of CNT could boost the synergistic effect of the nanocomposite by reducing the electron transfer resistances and prolonging the lifetime of electron-hole pairs. However, as for AgSiOx@RGO, adsorption effect is dominant rather than photodegradation as RGO contains residual oxygen-containing groups.

Yang et al. presented a comparative study of photocatalytic selective oxidation on several carbon based photocatalysts (Yang M. Q. et al., 2013). They synthesized a series of TiO_2_-GR, –CNT, and –C_60_ photocatalysts by combining sol-gel with hydrothermal methods. These three different carbon allotropes affected slightly in the morphology, crystal phase, particle size, pore volume and surface area the of the supported TiO_2_ nanocrystals. The TiO_2_-carbon (GR, CNT, and C_60_) have similar photocatalytic activities and analogous reaction mechanisms toward selective oxidation of benzyl alcohol. Different preparation methods could obtain different structural composition and synergetic interaction between TiO_2_ and carbon, which therefore have a greater impact on the photocatalytic performance of TiO_2_-carbon composites. The comparison shows that GR fails to prove its unique advantage compared to the other two carbon allotropes. Similarly, Zhang et al. investigated TiO_2_-Graphene as high-performance photocatalyst for the gas-phase degradation of benzene (Zhang et al., 2010). They concluded that GR was in essence the same as other carbon materials (carbon nanotube, activated carbon, and fullerene) as cocatalysts on enhancement of photocatalytic activity of TiO_2_, although GR has unique structural and electronic properties in comparison with other carbon allotropes.

Due to the special 2D structure and excellent physical/chemical properties, we expected the graphene will show better performance compared to other carbon allotropes (An and Yu, 2011; Zhang et al., 2011). However, it didn't show superior cocatalytic properties compared to the CNT or carbon quantum for the modification of some semiconductors (Ma et al., 2016). Researchers has tried to modify the graphene further by heteroatoms doping or activation method, which could increase its electric conductivity or surface area. The performance of the modified graphene could be then enhanced further as photocatalytic cocatalysts, thus increasing its real application potential.

## Mechanism of carbon cocatalysts for photocatalytic activity improvement

It has been proposed that the photocatalytic activity enhancement is due to the synergistic effect between semiconductor and carbon materials. Generally, carbon materials play four primary roles as cocatalysts for the activity enhancement of the semiconductors (Tan et al., 2012; Bai et al., 2016). (1) They provide a structure with larger specific surface area over which the active component can be well-dispersed, thus increasing the active sites. Activated carbon is amorphous carbon with a specific surface up to 3,000 m^2^ g^−1^ (Strobel et al., 2006). Graphene, the 2-dimensional nanosheets composed of sp^2^-hybridized carbon atoms, possesses an extremely high specific surface area (theory value of 2,630 m^2^ g^−1^) (Fan X. et al., 2015). While the CQDs can distribute uniformly on the surface of semiconductor materials because of its small size. (2) During the photocatalytic degradation of organic pollutants, carbon materials can be used as adsorbent to improve the adsorption capacity of semiconductors (Matos et al., 2001; Ai et al., 2015). (3) Carbon materials can be doped as a photosensitizer for bandgap narrowing, which is favorable for expanding the visible light absorption region of semiconductors. (4) By the formation of carbon materials–semiconductor heterojunction, the excellent electron transfer could be achieved, leading to the enhanced charge separation efficiency and photocatalytic activity (Guldi et al., 2006; Li X. et al., 2016; Shi et al., 2017).

## Conclusions and future prospects

Carbon materials are important photocatalytic cocatalysts due to their low cost and high efficient. In this review, we summarized the recent development of the carbon materials based semiconductor photocatalysts, including their synthesis methods and the applications for H_2_ evolution and pollutants degradation. Zero-dimensional C_60_, CQDs, one-dimensional CNTs, two-dimensional GR, and activated carbon are all involved to provide valuable information for metal free cocatalysts selection. Although much progress has been achieved, some essential issues are still unaddressed, especially for the activity and stability enhancement mechanisms. Studies about the interface between the semiconductors and the cocatalysts should be helpful for new carbon materials based photocatalysts development. Computational chemistry using DFT could also provide valuable information for the photocatalysts design. Although more in-depth studies are still needed, carbon materials based photocatalysts have great potential to address various environmental and energy-related problems.

## Author contributions

WH chose the references and edit the draft. ZL provided assistance for literature search and some revision. YL, XF, FZ, and GZ provided professional advice. WP designed the main content and revised the manuscript. All authors read and approved the final manuscript version to be submitted.

### Conflict of interest statement

The authors declare that the research was conducted in the absence of any commercial or financial relationships that could be construed as a potential conflict of interest.
